# Leveraging blood-based transcriptomics to detect acute cellular rejection in lung transplant

**DOI:** 10.1016/j.jhlto.2024.100081

**Published:** 2024-03-04

**Authors:** Auyon J. Ghosh, Matthew Moll, Shikshya Shrestha, Sergio Poli, Stephen J. Glatt, Hilary J. Goldberg, Andrew M. Courtwright, Souheil Y. El-Chemaly

**Affiliations:** a Division of Pulmonary, Critical Care, and Sleep Medicine, SUNY Upstate Medical University, Syracuse, NY; b Channing Division of Network Medicine, Brigham and Women’s Hospital, Boston, MA; c Division of Pulmonary and Critical Care Medicine, Brigham and Women’s Hospital, Boston, MA; d Harvard Medical School, Boston, MA; e Section on Pulmonary, Critical Care, Allergy, and Sleep Medicine, Veterans Affairs Boston Healthcare System, Boston, MA; f Department of Psychiatry and Behavioral Sciences, SUNY Upstate Medical University, Syracuse, NY; g Department of Pulmonary and Critical Care Medicine, Perelman School of Medicine, University of Pennsylvania, Philadelphia, PA

**Keywords:** transcriptomics, acute cellular rejection, lung transplant, chronic lung allograft dysfunction, biomarkers

## Abstract

Acute cellular rejection (ACR) is one of the main risk factors for chronic allograft dysfunction, the primary contributor to poor long-term survival in lung transplant recipients. We sought to develop a blood-based transcriptomic risk score (TRS) to detect ACR in lung transplant recipients. We tested for the association of the TRS with ACR in a logistic mixed model. We analyzed 101 samples from 75 individuals. We identified 4 genes after application of the least absolute shrinkage and selection operator. The TRS was significantly associated with ACR (odds ratio (OR) 3.43, 95% confidence interval (CI) 1.86-7.14, *p* < 0.001). The TRS demonstrates robust discrimination between the 2 groups given an area under receiver operator curve above 0.8, which could lead to less invasive diagnosis of ACR and prediction of individuals at risk for ACR in the future. Further studies with larger sample size are needed to firmly establish the clinical utility of the TRS for ACR.

## Background

Long-term survival in lung transplant recipients is primarily limited by chronic lung allograft dysfunction (CLAD).^1^ One of the main risk factors for development of chronic lung allograft dysfunction is acute cellular rejection (ACR).^2^ Transbronchial biopsy (TBBx) remains the gold standard for obtaining lung tissue for evaluation of ACR; however, the risks of complications or inadequate sampling may be limiting.^3^ There is substantial interest in identifying biomarkers in more-readily available tissues that can successfully diagnose and predict the development of ACR. This includes quantifying cell-free DNA and analyzing the cell pellet produced from bronchoalveolar lavage.^4^, ^5^, ^6^ Blood-based transcriptomics offer an attractive option for further study given low costs and promise in several other complex lung diseases.^7^ We hypothesized that a blood-based transcriptomic risk score (TRS) could detect ACR in lung transplant recipients.

## Material and methods

Participants were recruited from the Brigham and Women’s Hospital (BWH) Lung Transplant program from July 1, 2015, to December 31, 2017. The Brigham and Women’s Hospital Institutional Review Board approved the study (IRB #2013P000657) and all participants provided written informed consent. ACR was defined by the presence of perivascular mononuclear infiltrates assessed by a pathologist on TBBx samples. Blood was collected from recipients undergoing TBBx for ACR surveillance. mRNA was purified from total RNA using poly-T oligo-attached magnetic beads. The NEBNext Ultra II RNA Library Prep Kit by Illumina was used for library preparation. Libraries were quantified using Qubit and sequenced on the NovaSeq6000 S4 Illumina. Paired-ended FASTQ files were aligned to a reference genome (hg38) using STAR 2.5.2b.

Prior to analysis, genes with <1 count per million reads in more than 50% of samples were excluded. We performed differential expression analysis using the *limma* and *voom* R packages, and tested associations between gene expression levels and ACR. Models were adjusted for age, race, sex, and cytomegalovirus (CMV) donor/recipient mismatch status. We accounted for intrasubject correlations for samples obtained from the same individual using the duplicateCorrelation function in *limma.*^8^ We adjusted for multiple testing with a false discovery rate <10%. We performed pathway enrichment analyses using the Gene Ontology (GO) reference pathways. We applied a penalized regression framework with the *glmnet* package to develop a TRS for ACR. The TRS development framework has been previously published.^9^ Briefly, we used a least absolute shrinkage and selection operator to shrink coefficients toward zero and automate feature selection. For TRS development, only the sample most proximal to transplant was included for participants with multiple samples. We tested for association of the TRS with ACR using all available samples in a logistic mixed model adjusted for time since transplant, age, race, sex, CMV mismatch status, lifetime development of de novo donor-specific antibodies, and number of human leukocyte antigen (HLA) mismatches.

## Results

We obtained RNA sequencing data from 150 samples from 96 individuals. Forty-seven samples were excluded due to inconclusive pathologic assessment (i.e., AX) and 2 samples were excluded for poor RNA quality. We analyzed 101 samples from 75 individuals. ACR was present in 21.8% of samples (22/101), and the majority (72.7%) of ACR samples had A-grade rejection. We compared control and ACR samples by demographics and clinical characteristics. There were no differences in demographics, CMV mismatch status, or time since transplant between the 2 groups (Table 1).

**Table 1:** Subject Characteristics

Characteristic	Control	ACR	*p*-value
*n*	79	22	
Age, years	56.1 (11.7)	57.1 (8.6)	0.712
Female sex (%)	30 (38.0)	5 (22.7)	0.282
Non-White race (%)	8 (10.1)	3 (13.6)	0.936
CMV donor/recipient mismatch status (%)	29 (36.7)	9 (40.9)	0.912
Time since transplant, days	177.3 (133.0)	149.4 (121.8)	0.378
Lifetime dnDSA (%)	8 (11.4)	1 (4.8)	0.631
Number of HLA mismatches	5.00 [4.00, 5.00]	5.00 [4.00, 5.00]	0.323
A-grade rejection (%)			<0.001
A0	79 (100.0)	3 (13.6)	
A1	0 (0.0)	7 (31.8)	
A2	0 (0.0)	9 (40.9)	
AX	0 (0.0)	3 (13.6)	
B-grade rejection (%)			<0.001
B0	64 (81.0)	10 (45.5)	
B1	0 (0.0)	6 (27.3)	
B2	0 (0.0)	1 (4.5)	
BX	15 (19.0)	5 (22.7)	
TRS (scaled)	-0.23 (0.88)	0.84 (0.99)	<0.001

Abbreviations: ACR, acute cellular rejection; CMV, cytomegalovirus; dnDSA, donor-specific antibodies; HLA, human leukocyte antigen; TRS: transcriptomic risk score.

Data presented as mean (SD), median [IQR], or no. (%).

We identified 23,661 genes among all eligible samples. After filtering for low-expressed genes, we examined 12,900 genes for differential expression between ACR and control samples. We identified 110 genes that were differentially expressed with a false discovery rate of 10%. We then performed pathway enrichment using the differentially expressed gene set annotated with the GO Biological Process, Molecular Function, and Cell Component terms. There were no GO Molecular Function pathways that were significantly enriched (adjusted *p* < 0.05) and only 5 GO Cell Component pathways that were enriched. In contrast, there were 27 GO Biological Process pathways, primarily immune-related, that were significantly enriched, including antibacterial humoral response, innate immune response in mucosa, lymphocyte chemotaxis and migration, and steroid hormone-mediated signaling pathway.

**Figure 1:**
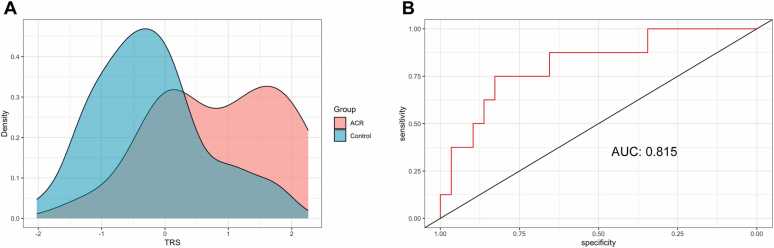
Distribution of TRS and receiver operator characteristic curve. (A) Density plot demonstrating the distribution of the TRS between ACR samples (orange) and control samples (blue). (B) Receiver operator characteristic curve for prediction of ACR. ACR, acute cellular rejection; AUC, area under receiver operator curve; TRS, transcriptomic risk score.

To develop the TRS, we used 10-fold cross-validation to identify the lambda (λ = 0.1111548) that minimized mean squared error. After application of the lambda, we identified 4 genes, *DICER1-AS1*, *ZNF426*, *TRIM56*, and *DEFA1B*, all of which were present in the differentially expressed gene set. We computed the TRS for each sample by summing the log-transformed counts of the 4 genes, weighted by the coefficients determined by the model. The scaled distribution of the TRS between ACR and control samples is shown in Figure 1A. In an adjusted logistic mixed model, the TRS was significantly associated with ACR (odds ratio (OR) 3.43, 95% CI 1.86-7.14, *p* < 0.001). None of the other covariates were significantly associated with ACR. After splitting the dataset into training (*n* = 64) and testing (*n* = 37) sets, the area under receiver operator curve (AUC) for the TRS was 0.815 (Figure 1B).

## Discussion

In this study of individuals who have received a lung transplant comparing samples with and without ACR as determined by TBBx, we show that blood-based gene expression reveals primarily immune-related differences between ACR and controls. We also developed a TRS that is associated with ACR, independent of several important clinical characteristics. We demonstrate that the blood gene-expression shows important, biologically plausible differences between ACR and control samples. For instance, *ZNF426* expression has previously been shown to be altered by calcineurin inhibitors.^10^ In addition, the TRS demonstrates robust discrimination between the 2 groups given an AUC above 0.8, which could lead to less invasive diagnosis of ACR and prediction of individuals at risk for ACR in the future. The primary limitation of our study is the lack of an independent replication cohort for our findings. Our sample size was limited due to the small pool of transplant recipients and the loss of samples due to indeterminate pathology. Future studies with larger sample size, and potentially pre- and post-ACR development samples, and ability to adjust for other parameters, such as underlying lung disease leading to transplant, immunosuppression and infectious status, are needed to firmly establish the clinical utility of the TRS for ACR and to evaluate the relationship between TRS and severity of rejection. Our findings open the door to improving the detection of ACR and ultimately improving lung transplant-related outcomes.

## CRediT authorship contribution statement

Concept and design: A.J.G., A.M.C., S.E.C.; Data collection: S.S., S.P., H.J.G., A.M.C., S.E.C.; Data analysis: A.J.G., M.M., S.S., S.P., S.E.C.; Statistical support: A.J.G., M.M., S.P., S.E.C.; all authors were responsible for critical revision of the manuscript for important intellectual content.

## Disclosure statement

The authors declare that they have no known competing financial interests or personal relationships that could have appeared to influence the work reported in this paper.

A.J.G. is supported by K08HL168205. M.M. is supported by K08HL159318. S.J.G. is supported by R01AG064955. S.E.C. is supported by the John M. Memorial Fund. S.E.C. was an employee of Sanofi at the time of publication. S.S. was an employee of Vertex Pharmaceuticals at the time of publication.

## Data Availability

The RNA-Seq data supporting the findings of this study are available in GEO DATABASE at https://www.ncbi.nlm.nih.gov/geo/.
